# Movement Estimation Using Soft Sensors Based on Bi-LSTM and Two-Layer LSTM for Human Motion Capture

**DOI:** 10.3390/s20061801

**Published:** 2020-03-24

**Authors:** Haitao Guo, Yunsick Sung

**Affiliations:** Department of Multimedia Engineering, Dongguk University-Seoul, Seoul 04620, Korea; haitao@dongguk.edu

**Keywords:** human motion capture, movement estimation, HTC VIVE, Myo armband, soft sensor

## Abstract

The importance of estimating human movement has increased in the field of human motion capture. HTC VIVE is a popular device that provides a convenient way of capturing human motions using several sensors. Recently, the motion of only users’ hands has been captured, thereby greatly reducing the range of motion captured. This paper proposes a framework to estimate single-arm orientations using soft sensors mainly by combining a Bi-long short-term memory (Bi-LSTM) and two-layer LSTM. Positions of the two hands are measured using an HTC VIVE set, and the orientations of a single arm, including its corresponding upper arm and forearm, are estimated using the proposed framework based on the estimated positions of the two hands. Given that the proposed framework is meant for a single arm, if orientations of two arms are required to be estimated, the estimations are performed twice. To obtain the ground truth of the orientations of single-arm movements, two Myo gesture-control sensory armbands are employed on the single arm: one for the upper arm and the other for the forearm. The proposed framework analyzed the contextual features of consecutive sensory arm movements, which provides an efficient way to improve the accuracy of arm movement estimation. In comparison with the ground truth, the proposed method estimated the arm movements using a dynamic time warping distance, which was the average of 73.90% less than that of a conventional Bayesian framework. The distinct feature of our proposed framework is that the number of sensors attached to end-users is reduced. Additionally, with the use of our framework, the arm orientations can be estimated with any soft sensor, and good accuracy of the estimations can be ensured. Another contribution is the suggestion of the combination of the Bi-LSTM and two-layer LSTM.

## 1. Introduction

Recently, the demand for human movement estimation based on soft sensors has increased in the field of human motion capture. Human motion is widely utilized for the natural user interface/experience (NUI/NUX) in humanized computing environments [1,2,3], which needs advanced technology of human motion capture and estimation. Two kinds of sensory devices are developed for capturing motion: graphical data-based devices and time series data-based devices. 

Graphical data-based devices provide a means for end-users to interact with computers with the aid of one or more cameras. One typical graphical data-based sensory device is the Microsoft Kinect motion-sensing input device [4]. Kinect is popular because it creates a novel way for end-users to interact with computers. End-users can control the virtual characters directly through their body movements, without any other attached sensor [5,6]. However, it is difficult for Kinect to estimate subtle movements, particularly the movements that need sufficient operational and smooth sensory feedbacks.

Time series data-based sensory devices provide a means for end-users to interact with computers using one or more sensor-based controllers. HTC VIVE [6] is a powerful time series data-based sensory device (consisting of one headset and two controllers) that has been developed as a naturally interacting system. Users’ head and hand positions can be estimated accurately with the headset and controllers as the latter are directly measured by sensors in the former. Therefore, HTC VIVE is better suited to capture motions with accurate control [7,8].

The time series data-based sensory devices have limitations in capturing the end-user’s expression, such as the movement of arms and legs. To overcome this, multiple sensors can be attached to the user’s limbs [9,10] to enable accurate measurement. However, this makes it inconvenient for the end-users to move, and the collected sensory movements may be unnatural. Therefore, it is preferable to estimate the arm and leg movements with soft sensors. 

Previously, the arm movements were estimated based on Bayesian probability [11]. One HTC VIVE controller was utilized to collect the sensory value including the hand positions, and one Myo gesture-control sensory armband (Myo armband) was attached to an arm to collect its orientation. Bayesian probabilities were then calculated, considering the movements of the hand and arm. Arm movements were estimated by the corresponding movements of the highest Bayesian probabilities. However, for calculating Bayesian probability, estimative movements should be defined in advance. As there has been much research on the diverse kinds of domains applicable to deep learning networks [12,13], it is preferable that this method can estimate movements without the need for predefining movements in advance.

This paper proposes a framework to estimate the orientations of a paired upper arm and forearm of a single arm using a two-stream bidirectional two-layer long short-term memory (LSTM)-based framework (TBTLF), based on two-stream bidirectional two-layer long short-term memory (LSTM) fusion by combining Bi-LSTM and two-layer LSTM. Using Bi-LSTM, multiple consecutive sensory movements obtained from sensors can be analyzed. Using two-layer LSTM, high-level features can be analyzed, which also increases the accuracy of estimated movements. Using the proposed framework, sensory movements can be estimated without relying on pre-defined motions, thereby making the motion estimation process more flexible as well.

This paper is organized as follows: Section 2 introduces related works. Section 3 presents the proposed movement estimation framework. Section 4 validates the proposed framework experimentally and compares its performance with the traditional framework. Section 5 discusses the issues and limitations associated with the proposed framework and Section 6 presents the conclusions of the study.

## 2. Related Works

Recently, movement estimation has been widely studied in human motion capture fields. This chapter introduces Bayesian-based and deep learning-based approaches for movement estimation.

### 2.1. Bayesian-Based Movement Estimation

Due to the current substantial cost of wearable sensors, it is desirable to reduce the number of sensors required to estimate movements. Some traditional algorithms, such as Bayesian probability and K-means, are utilized to estimate the movements of the unmonitored parts of a body by considering the movements measured directly using sensors [14]. Bayesian probability was first used to estimate the arm movement by Kim et al. [15]. Arm movements were measured using two Myo armbands (Thalmic Labs) and the estimations were presented as coordinate values of arm orientations. As the measured data differed among themselves despite presenting the same orientation, they were sorted into angles ranging from −180° to 180° at 30° intervals. The upper arm movement was estimated based on the maximum Bayesian probability between the movement orientation angles of the forearm and upper arm. Therefore, the movement of one arm (an upper arm and a forearm) was represented by one Myo armbands instead of two. This Bayesian probability-based approach was then improved by Lee et al. [16] using a MinMax movement estimation framework. In this approach, rather than using a fixed angle range of −180° to 180°, the angle range was determined by the minimum and maximum values of the measured data and thereby provided more accurate movement estimation.

Choi et al. proposed a Bayesian probability approach to estimate forearm orientations based on hand positions [11]. Forearm orientations were still measured by Myo armband, while the hand positions were collected using VIVE controllers. The unmeasured orientations of a forearm were estimated using the measured positions of a hand and the calculated Bayesian probability between the orientations of the forearm and the positions of the hand.

Bayesian-based approaches perform well for movement estimation with pre-defined motions. In such approaches, large amounts of data are collected using sensor-based wearable devices. However, only a small proportion of these data match pre-defined motions. Consequently, these rich data sets do not provide any benefits for improving the performance of movement estimation using Bayesian-based movement estimation approaches. However, deep learning has recently been widely used in many domains due to its excellent capability to deal with large amounts of data, and thereby offers an enhanced method for improving the performance of movement estimation.

### 2.2. Deep Learning-Based Movement Estimation Approaches

Technological improvements enable large amounts of movement data to be analyzed. Deep learning is the most popular approach for dealing with large amounts of data for movement estimation.

State-of-the-art performances have been reported in many human motion capture tasks based on deep learning algorithms [17]. One previous study proposed a deep neural network (DNN)-based framework to accurately estimate 3D poses from multi-view images [18].

MoDeep, developed by Arjun et al. [19], is a deep learning framework for estimating the two-dimensional (2D) locations of human joints based on the movement features in a video. A convolutional network architecture deals with color and movement features based on a sliding-window architecture. The input is a three-dimensional (3D) tensor, which is a combination of an RGB image and its corresponding movement features in optical flow, and the output is a 3D tensor comprising one response map for each joint.

Aria et al. [20] trained a Convolutional Neural Network (CNN) for the estimation of unsupervised movements. The input for this network is a pair of images, and a dense motion field can be produced at its output layer. This network is a fully convolutional neural network with 12 convolutional layers that could be regarded as two parts. In the first part, CNN makes a compact representation of the movement information, which involves four down samplings. In the second part, the compact representation is used to reconstruct the motion field; this involves four upsamplings. Then, the movement of the motion can be estimated.

However, MoDeep estimated human poses using the FLIC-motion dataset [21], which comprises 5003 images collected from Hollywood movies, augmented with movement features. Aria et al. trained a CNN using pairs of consecutive frames from the UCF101 dataset [22]. Both these two approaches estimated movements based on the visual information of human movements contained in the video. The goal of these approaches was to estimate the movements in the video frame sequences. For using the sensory data, Hu et al. [23] proposed a method to investigate the performance of the deep learning network with long short-term memory (LSTM) units to deal with the sensory value of an inertial motion unit (IMU). They verified that machine-learning approaches are able to detect the surface conditions of the road and age-group of the subjects from the sensory data collected from the walking behavior of the subjects. Therefore, a deep learning network should be proposed for estimating the movement based on the sensory movement values measured by wearable devices.

### 2.3. Comparison of the Bayesian-Based and Deep Learning-Based Movement Estimation

The Bayesian-based and deep learning-based movement estimation methods mentioned above are analyzed and compared with the framework proposed in this paper in Table 1.

From Table 1, it can be seen that there are mainly two types of conventional and widespread motion capture methods. These methods can be classified into image-based methods [18,19,20], which estimate the movement based on convolutional neural networks (CNNs) [23], and sensor-based methods, which use Bayesian probability [11,14,15] and LSTM [23]. In [11,14,15], the movements were estimated using Bayesian probability, whereas in [23], the surface conditions of the road and age-group of the subjects were detected based on the sensor values and an LSTM network. Owing to the significant contribution of deep learning methods in the field of motion capture, this study is expected to bring forth a deep learning-based framework, instead of the traditional methods [11,14,15], to improve the performance of VR applications using soft sensors. 

### 2.4. Consideration of Deep Learning Frameworks

This section introduces the most commonly used deep learning frameworks. A convolutional neural network was first designed for image recognition. A traditional CNN comprises three structures: convolution, activation, and pooling. The output of the CNN is the specific feature space of each image. CNN deals well with the image inputs due to its excellent ability in extracting the spatial features of the inputs. However, it is not widely used to deal with time-related sequence data.

Another popular deep learning neural network is the recurrent neural network (RNN) [24]. Compared with CNN, RNN provides better advantages in the processing of time-related sequence information, but its training architecture causes long-term dependency problems.

LSTM is used to solve the issue of long-term dependency through its special cell structure with several gates [25]. Like RNN, LSTM retains the ability to deal with long-term sequence data; however, only data before the current time can be used to train its relative parameters. Therefore, bidirectional LSTM (Bi-LSTM) [26] is used, because it has an excellent ability to process two-directional data. In traditional LSTM, the state of the LSTM cell is transmitted forward to backward, while in bidirectional LSTM, the outputs of the current time are decided considering not only the previous states but also the subsequent ones. Traditional Bi-LSTM contains two LSTM layers: forward LSTM layer and backward LSTM.

The method proposed in this paper is useful for dealing with the time-related sequence sensory data, which are collected by HTC VIVE controllers and Myo armbands. Each single layer (forward LSTM layer and backward LSTM layer) of a traditional Bi-LSTM can only utilize the primitive features of inputs. For estimating the movements of a single arm, the high-level features can be utilized to improve the accuracy of the estimated results. Therefore, the framework proposed herein adds a two-layer LSTM as a sub-layer of the Bi-LSTM to enhance the ability of expression for the entire learning model.

## 3. Movement Estimation Framework

The proposed framework estimates the orientations of a single arm, comprising a pair of an upper arm and a forearm, according to the movements of two hands (left and right hands). This chapter provides an overview of the movement estimation processes and the structure of TBTLF.

### 3.1. Overview

TBTLF is realized based on the newly proposed two-stream bidirectional two-layer LSTM (TBTL). TBTL is a combination of Bi-LSTM [26] and two-layer LSTM [27] and is built to deal with sensory movements, which are defined as those represented by combinations of sensory values.

**Figure 1:**
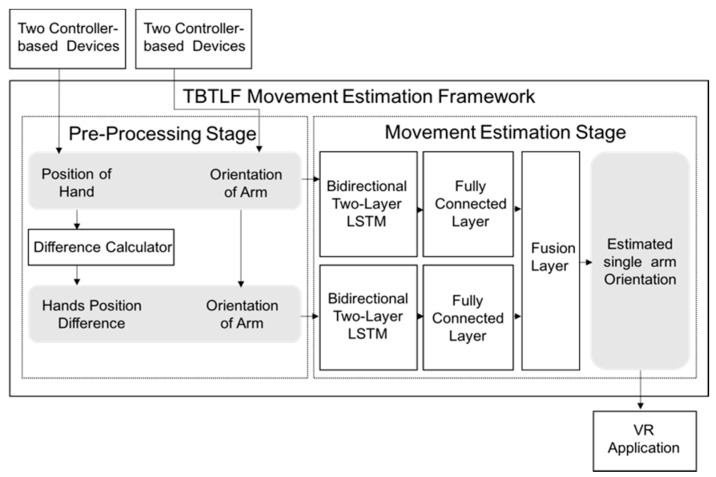
Overview of the proposed framework.

The proposed framework comprises two stages as shown in Figure 1: pre-processing and movement estimation. In the pre-processing stage, the positions of the left and right hands and the orientations of one arm are collected by two time-series-data-based devices and two gesture-based devices, respectively. The proposed framework in the movement estimation stage contains a two-stream architecture with bidirectional two-layer LSTMs and fully connected layers. Finally, the outputs of these two streams are combined with a fusion layer, and the fused outputs are provided as the final estimated orientation of a single arm.

**Figure 2:**
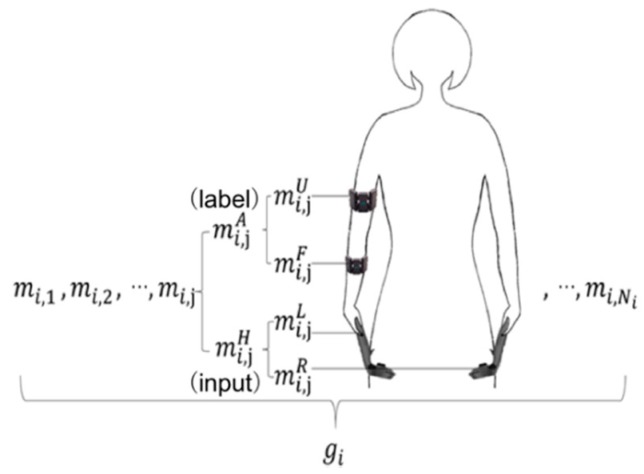
Locations of controllers and gesture-based devices.

A dataset was collected as the ground truth using two Myo armbands and two HTC VIVE controllers. The Myo armbands measure the orientations of an upper arm and a forearm, and the HTC measures the locations of the two hands. Subsequently, the proposed framework was used to estimate the orientations of a single arm, which could either be a left arm or right arm. An example of the placement of the two Myo armbands and two HTC VIVEs is shown in Figure 2. However, if two other Myo armbands are placed on the not-attached arm to collect the data of the corresponding arm, the orientations of both the left arm and right arm can be estimated by training the proposed framework twice using the left-arm dataset and right-arm dataset, respectively.

### 3.2. Pre-Processing Stage

The sensory movement, $m_{t}$, measured at time t by two-time series data-based devices and two gesture-based devices is defined by the sensory values of the pair of the arm movement $m_{t}^{A}$ and the hand movement $m_{t}^{H},$ as shown in Equation (1).

(1) $$m_{t}=\left[m_{t}^{A},m_{t}^{H}\right]$$

The arm movement $m_{t}^{A}$ consists of the upper arm movement $m_{t}^{U}$ and forearm movement $m_{t}^{F},$ as shown in Equation (2).

(2) $$m_{t}^{A}=\left[m_{t}^{U},m_{t}^{F}\right]$$

The upper arm movement $m_{t}^{U}$ and the forearm movement $m_{t}^{F}$ are defined as the corresponding orientations expressed by Equations (3) and (4), and they are measured by two gesture-based devices that collect the orientations as the motion quaternions (orientation coordinates, x, y, z, and w).

(3) $$m_{t}^{U}=\left[x_{t}^{U},y_{t}^{U},z_{t}^{U},w_{t}^{U}\right]$$

(4) $$m_{t}^{F}=\left[x_{t}^{F},y_{t}^{F},z_{t}^{F},w_{t}^{F}\right]$$

The hand movement $m_{t}^{H}$ is defined by the left-hand movement $m_{t}^{L}$ and the right-hand movement $m_{t}^{R},$ as shown in Equation (5).

(5) $$m_{t}^{H}=\left[m_{t}^{L},m_{t}^{R}\right]$$

The left-hand movement $m_{t}^{L}$ and the right-hand movement $m_{t}^{R}$ are defined as the positions from time series data-based devices, as shown in Equations (6) and (7).

(6) $$m_{t}^{L}=\left[x_{t}^{L},y_{t}^{L},z_{t}^{L}\right]$$

(7) $$m_{t}^{R}=\left[x_{t}^{R},y_{t}^{R},z_{t}^{R}\right]$$

The differences between the two hand positions obtained consecutively are used as the short-term information on the corresponding movement to improve the accuracy of the proposed framework. In this study, the difference in each hand position, $d_{t}^{H}=\left[d_{t}^{L},d_{t}^{R}\right]$, was calculated by a difference calculator. The left-hand position difference $d_{t}^{L}$ and the right-hand position difference $d_{t}^{R}$ are as shown in Equations (8) and (9), respectively.

(8) $$d_{t}^{L}=\left[d_{t}^{L,X},d_{t}^{L,X},d_{t}^{L,X}\right]$$

(9) $$d_{t}^{R}=\left[d_{t}^{R,X},d_{t}^{R,X},d_{t}^{R,X}\right]$$

where $d_{t}^{L,X}=x_{t}^{L,X}$-$x_{t-1}^{L,X}$ and so on. 

In the results, low-level features consist of the arm movement, hand movement, and hand position differences, where $l_{t}=\left[m_{t}^{A},m_{t}^{H},d_{t}^{H}\right]$. For training relative parameters, the arm movement $m_{t}^{A}$ and hand movement $m_{t}^{H}$ are used as inputs to the first stream of the TBTL, whereas $m_{t}^{A}$ and $d_{t}^{H}$ are used as the inputs to the second stream of the TBTL.

### 3.3. Movement Estimation Stage

**Figure 3:**
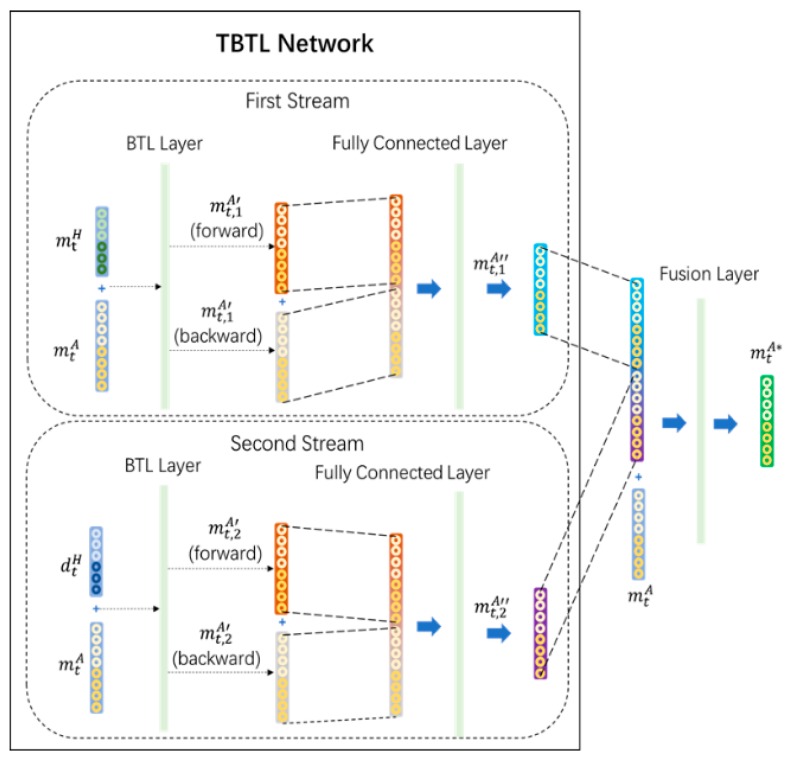
Structure of the two-layer long short-term memory (LSTM)-based framework (TBTLF) network.

The movement estimation stage includes two parts: a TBTL network and a fusion layer, as shown in Figure 3.

The proposed framework is based on two-stream structures. Given that a single bidirectional two-layer LSTM (BTL) stream is not able to capture the hierarchy of features in its entirety [26], another BTL is added to consider the hand position differences.

The differences between the hand positions provide the short-term movement features between two consecutive movements, which aid the estimation of movements by combining the advantage of Bi-LSTM for the long-term features of inputs with the advantage of the short-term movement features. 

**Figure 4:**
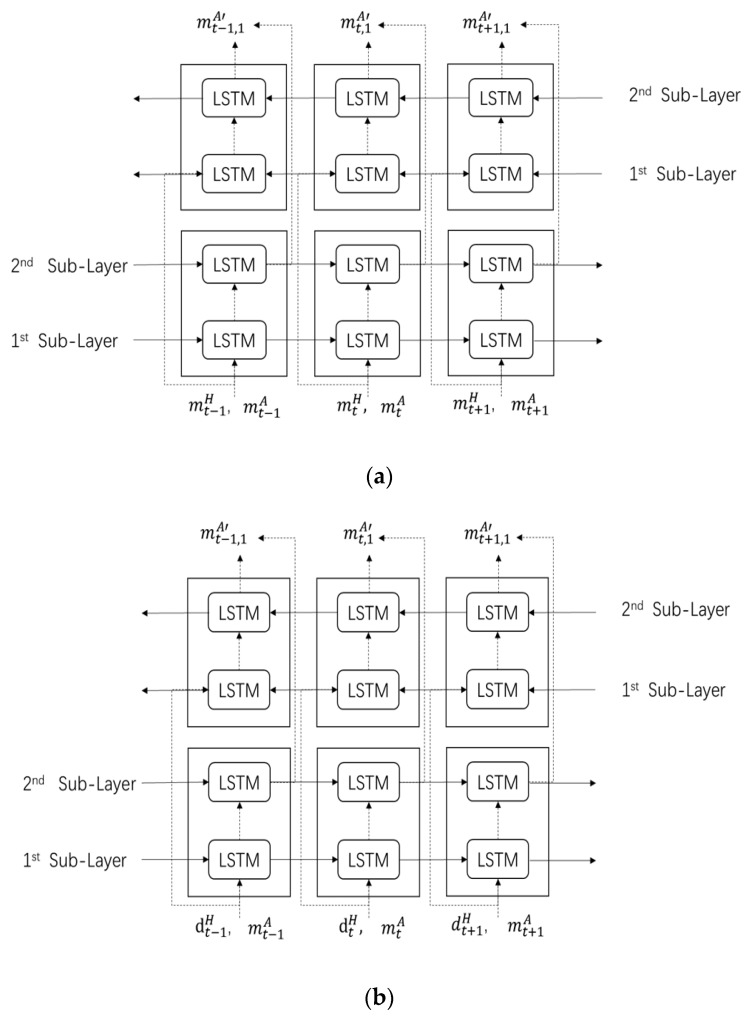
Structure of bidirectional two-layer LSTM (BTL) networks. (**a**) BTL network for the first stream in the TBTL network; (**b**) BTL network for the second stream in the TBTL network.

Two streams are applied to deal with low-level features. Then, two preliminary arm movements are estimated by the forward propagation and back-propagation of each BTL layer. The structures of the BTL for each stream in the TBTL are shown in Figure 4, considering time sequences. 

The two arm movements estimated by the TBTL network are concatenated and input to a fully connected layer. The secondary estimated arm movement is $m_{t,k}^{A^{\prime \prime }},$ as shown in Equation (10), and is generated by the *k*th stream.

(10) $$m_{t,k}^{A^{\prime \prime }}=\left[m_{t,k}^{U^{\prime \prime }},m_{t,k}^{F^{\prime \prime }}\right]$$

where $m_{t,k}^{U^{\prime \prime }}$ and $m_{t,k}^{F^{\prime \prime }}$ are the secondary estimated upper arm movements and forearm movements. They consist of the secondary estimated orientations of the upper arm and the forearm, as shown in Equations (11) and (12).

(11) $$m_{t,k}^{U^{\prime \prime }}=\left[x_{t,k}^{U^{\prime \prime }},y_{t,k}^{U^{\prime \prime }},z_{t,k}^{U^{\prime \prime }},w_{t,k}^{U^{\prime \prime }}\right]$$

(12) $$m_{t,k}^{F^{\prime \prime }}=\left[x_{t,k}^{F^{\prime \prime }},y_{t,k}^{F^{\prime \prime }},z_{t,k}^{F^{\prime \prime }},w_{t,k}^{F^{\prime \prime }}\right]$$

where $x_{t,k}^{U^{\prime \prime }},y_{t,k}^{U^{\prime \prime }},z_{t,k}^{U^{\prime \prime }},w_{t,k}^{U^{\prime \prime }}$ and $x_{t,k}^{F^{\prime \prime }},y_{t,k}^{F^{\prime \prime }},z_{t,k}^{F^{\prime \prime }},w_{t,k}^{F^{\prime \prime }}$ are the coordinates of the secondary estimated orientations of the upper arm and the forearm.

The secondary estimated arm movements of both streams are concatenated and input to a fusion layer, which is another fully connected structure. Therefore, the final estimated arm movement $m_{t}^{A^{*}}$ is generated as shown in Equation (13).

(13) $$m_{t}^{A^{*}}=\left[m_{t}^{U^{*}},m_{t}^{F^{*}}\right]$$

where $m_{t}^{U^{*}}$ and $m_{t}^{F^{*}}$ are the final estimated upper arm movement and forearm movement, respectively. They consist of the final estimated orientations of the upper arm and the forearm as shown in Equations (14) and (15).

(14) $$m_{t,k}^{U^{*}}=\left[x_{t,k}^{U^{*}},y_{t}^{U^{*}},z_{t}^{U^{*}},w_{t}^{U^{*}}\right]$$

(15) $$m_{t}^{F^{\prime \prime }}=\left[x_{t}^{F^{*}},y_{t}^{F^{*}},z_{t}^{F^{*}},w_{t}^{F^{*}}\right]$$

where $x_{t,k}^{U^{*}},y_{t}^{U^{*}},z_{t}^{U^{*}},w_{t}^{U^{*}}$ and $x_{t}^{F^{*}},y_{t}^{F^{*}},z_{t}^{F^{*}},w_{t}^{F^{*}}$ are the coordinates of the final estimated orientations of the upper arm and the forearm.

## 4. Experiments

### 4.1. Experimental Goals

The proposed framework focuses on the movement estimation of a single arm, which could either be a left arm or a right arm, including its corresponding upper arm and forearm, based on the positions of the two hands. In the experiments, since the positions of the two arms are required to be estimated, the experiments were repeated twice with the proposed framework: once for the right arm, and the second time for the left arm. The performance of the proposed framework was then compared with those of the Bayesian-based approach [11].

### 4.2. Experimental Environments

Two types of experiments were conducted. First, in the TBTLF-based experiments, movements were estimated by the proposed framework trained with 2000, 20,000, and 200,000 episodes. In these experiments, an episode is the time taken to repeat the training data during the training of the framework. Then, comparative trials based on the Bayesian-based movement estimation approach [11] were conducted with 50, 100, and 1000 intervals, respectively. In the Bayesian-based experiments, an interval is the number of subsections left after all the training data are divided uniformly [16]. Therefore, the best performances of each type of experiment were compared based on the distance calculated by dynamic time warping (DTW) [28], which is widely used to compare the similarity of two sequences.

All experiments were conducted on a computer running the Windows 10 Pro operating system with an Intel i7-7700 3.6 GHz processor, NVIDIA GeForce GTX-1050-2GB graphics card, and 16G of DDR4 RAM. Hand positions were measured with HTC VIVE controllers [7] and arm orientations were measure with two Myo armbands [14]. The dataset was collected by a Unity 3D project, which was developed based on HTC VIVE SDK (software development kit) and Myo SDK using C# programming language. All experiments were carried out using the python programming language based on the TensorFlow deep learning architecture. 

The ground truth of the proposed framework comprises the measured values of the two hand positions and single-arm orientations that are used for comparison with the estimated arm orientations. The ground truth was collected based on a VR game called “Rise of the Tomb Raider” [29]. 

**Figure 5:**
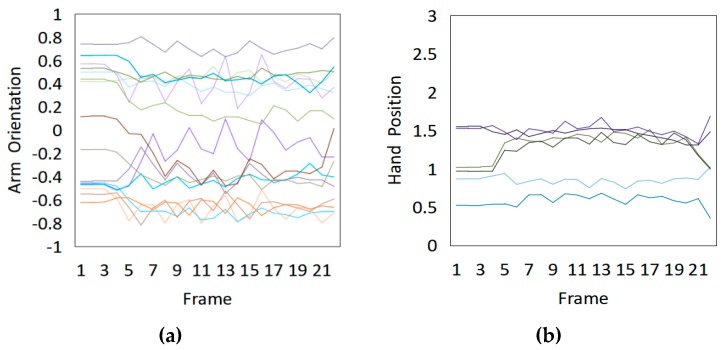
Collected arm orientations and hand positions for the running motion. (**a**) Arm orientations; (**b**) Hand positions.

**Figure 6:**
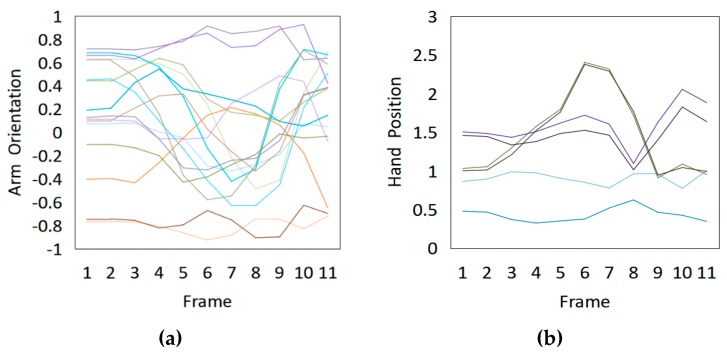
Collected arm orientations and hand positions for the jumping motion. (**a**) Arm orientations; (**b**) Hand positions.

Fifteen gestures represented by sensory values collected from two HTC VIVEs and two Myo armbands were used to train the proposed framework. The gestures in Table 2 are the gesture commands used for training and evaluation. The gestures were combined with several consecutive motions. There are 11 motions in total such as running, shooting, and jumping. Each motion is defined by multiple movements, consecutive combinations of the orientations of arms and the positions of hands. The collected arm orientation and hand position for running and jumping are shown in Figure 5 and Figure 6. Considering the playing of the game [29], the gestures are predefined.

Every motion was performed 10 times. Seven times of the performed motions (70%) were used as the dataset for training the proposed framework, which is referred to as the training data. Three times of the motions (30%) were used as the dataset for validating the proposed framework, which is referred to as the validation data. To demonstrate the performance of the proposed framework in experiments on different subjects, the data collected from three subjects were used to validate the proposed framework. The corresponding anthropomorphic information is shown in Table 3.

Both the training data and the validation data contained the measured arm orientations and hand positions measured simultaneously by Myo armbands and HTC VIVE controllers. The training data was used for training the parameters in the proposed framework. The measured hand positions in the validation data were used to generate the estimated arm orientations using the proposed framework or Bayesian-based framework [11], while the measured arm orientations were used to calculate the similarity to the estimated arm orientations by DTW. To train the TBTL network, several sets of hyper-parameters were adjusted. Finally, hidden_size was set to 256, time_steps to 160, and batch_size to 128.

### 4.3. Dataset Collection

**Figure 7:**
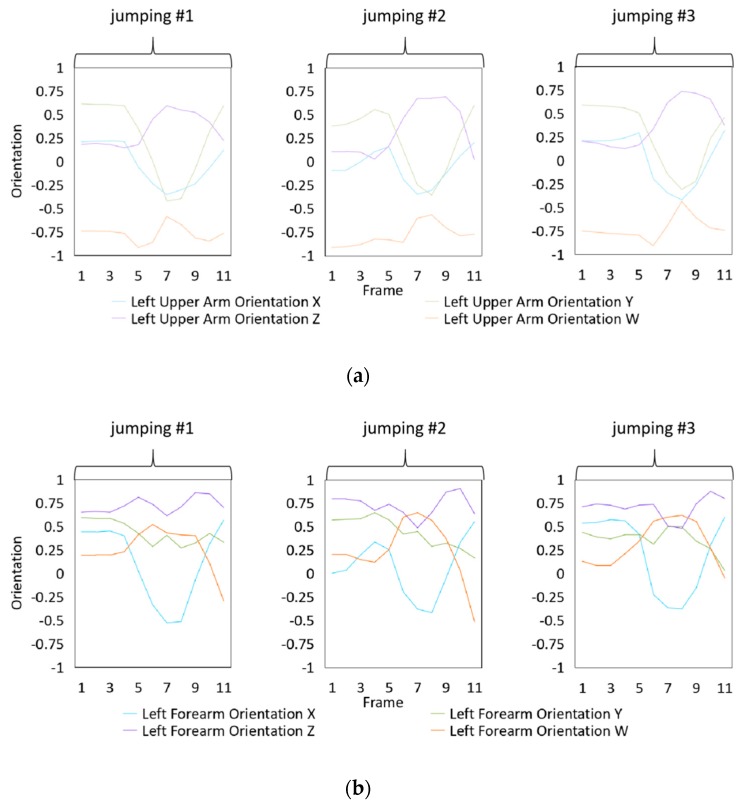
Dataset collected from the jumping motion of the left arm: measured orientations of the (**a**) Left upper arm and (**b**) Left forearm.

**Figure 8:**
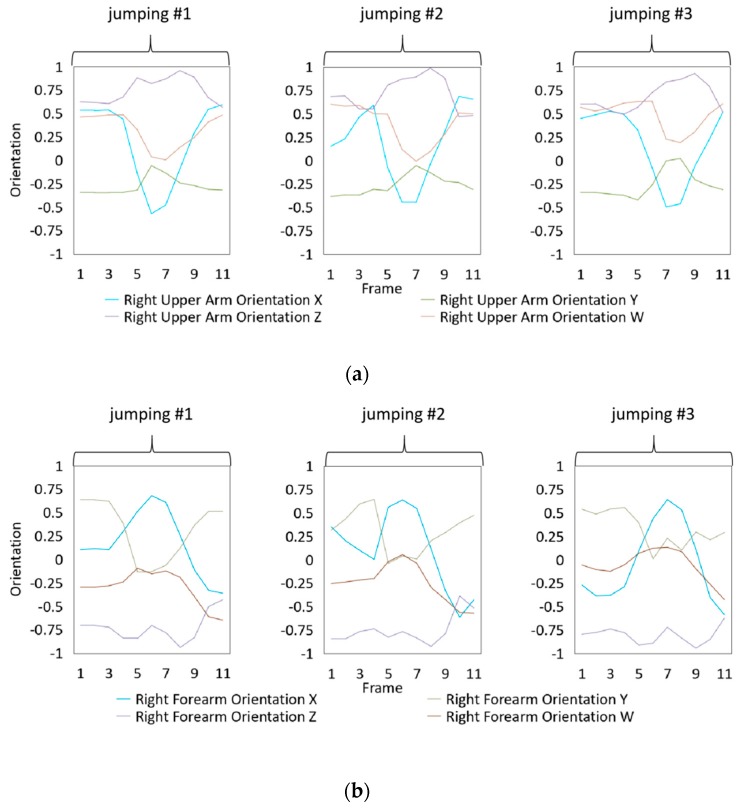
Dataset collected from the jumping motions of the right arm: measured orientations of the (**a**) Right upper arm and (**b**) Right forearm.

**Figure 9:**
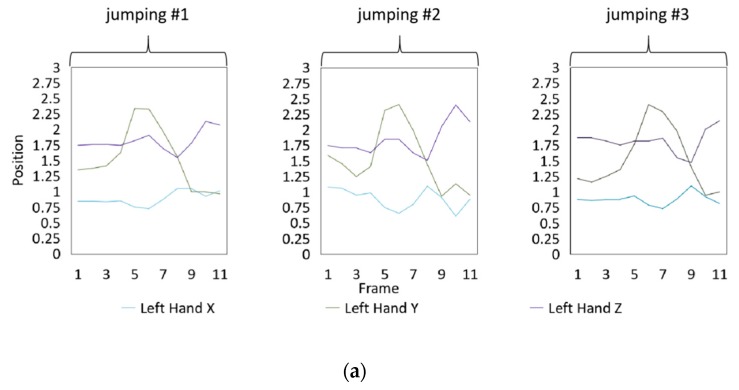

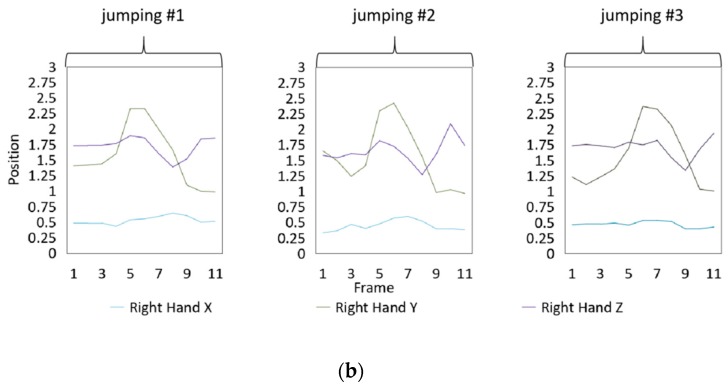
Training data of the measured positions of hands. Measured positions of the (**a**) Left hand and (**b**) Right hand.

To illustrate the performed motions, some of the data collected for jumping motions are shown in Figure 7, Figure 8 and Figure 9. The values in Figure 9 were used as the input of the proposed framework, and the those in Figure 7 and Figure 8 were used as the labels when training the frameworks for the left and right arm, respectively. In addition, they were also used as the ground truth to perform the evaluation experiments.

In these figures, *Frame* is defined to describe one set of data that was collected at the same time. *Orientation* is defined as the collected orientation of arm with a range of –1 to 1. *Position* is defined as the collected position of the hand, which is represented by the distance between base stations and controllers of HTC VIVE. 

### 4.4. Experimental Results

The measured data of the gestures with Indexes 1-15 was used to perform the evaluation experiments with its order as the ground truth. All gestures are performed by three subjects, one by one. Therefore, the ground truth data includes 11 motions, walking, running, picking up, shaking tree, jumping, avoiding, shooting, towing, opening door, sneaking, and attacking. 

**Figure 10:**
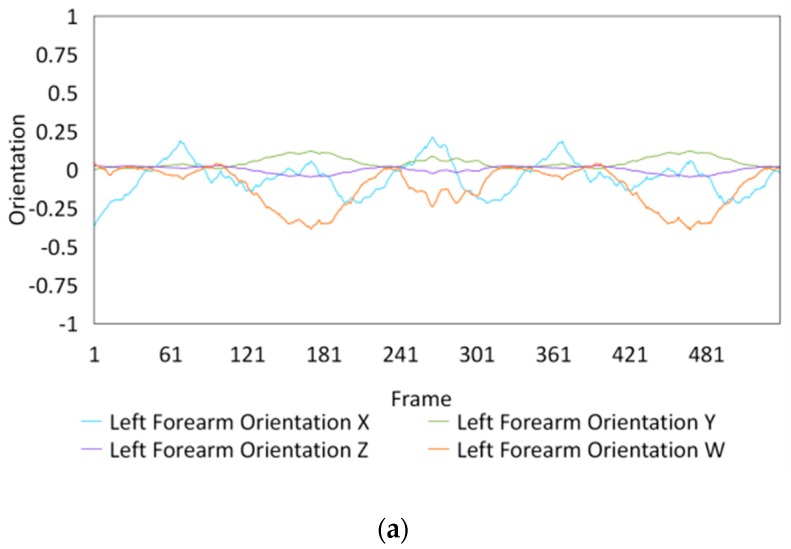

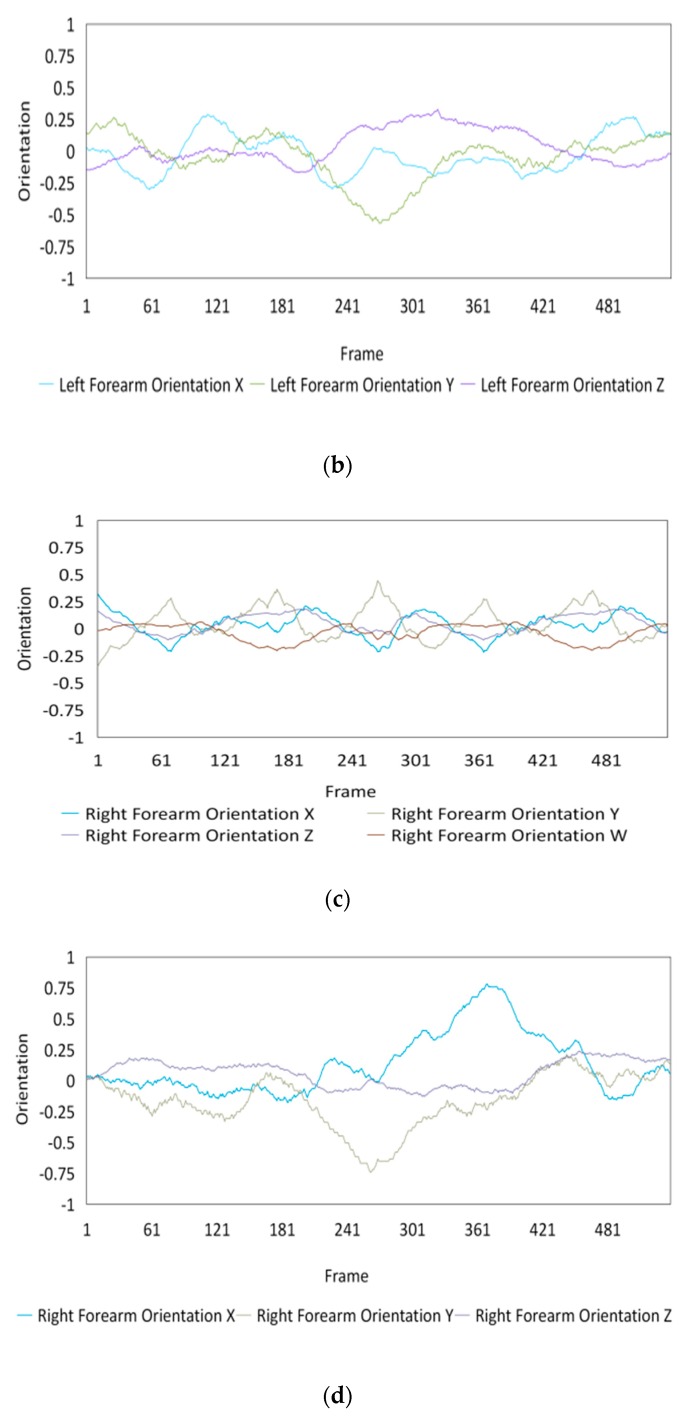
Comparison of the estimated orientations of the left arm for the 1st subject. (**a**) Left forearm with TBTLF-based experiments in 200,000 episodes. (**b**) Left forearm with Bayesian-based experiments with 50 intervals. (**c**) Right forearm with TBTLF-based experiments in 200,000 episodes. (**d**) Right forearm with Bayesian-based experiments with 50 intervals.

**Figure 11:**
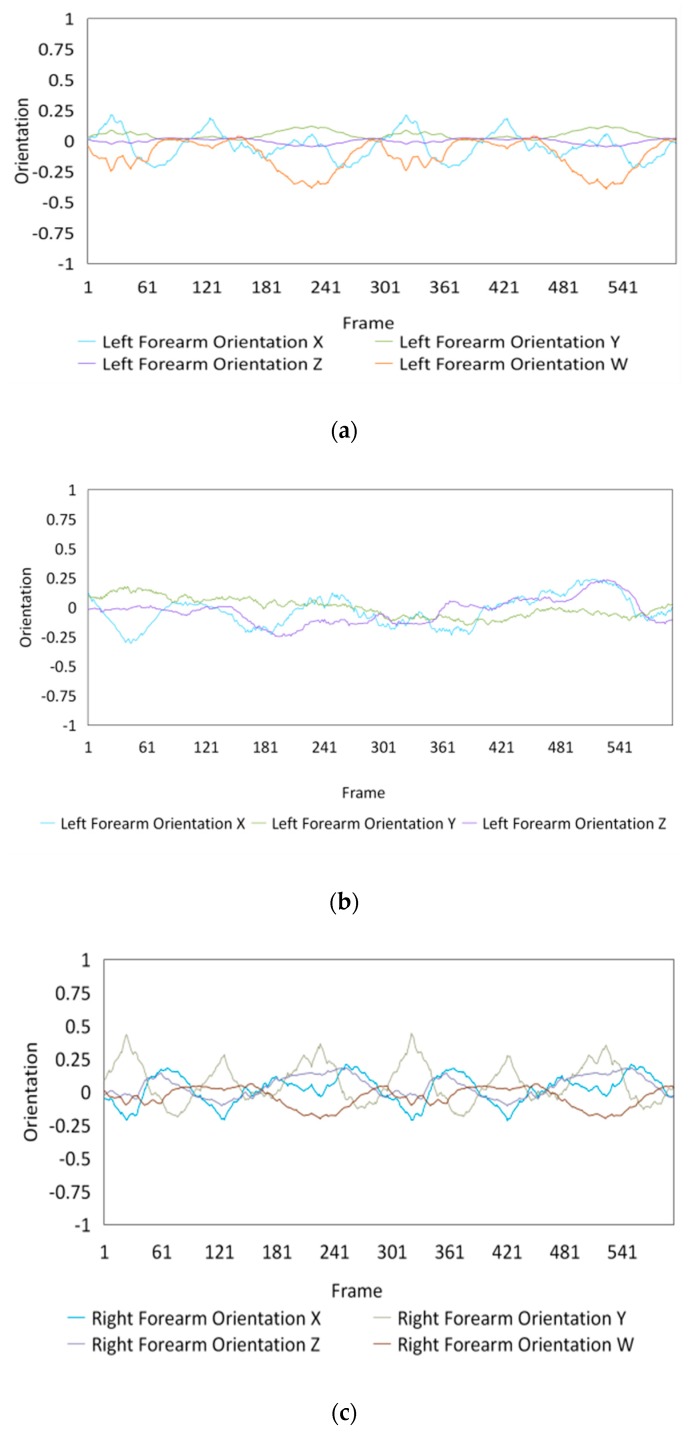

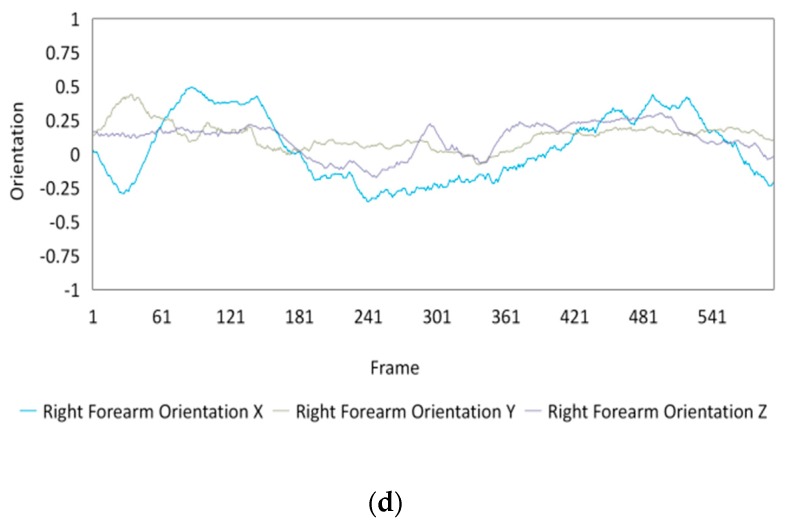
Comparison of the estimated orientations of the left arm for the 2nd subject. (**a**) Left forearm with TBTLF-based experiments in 200,000 episodes. (**b**) Left forearm with Bayesian-based experiments with 50 intervals. (**c**) Right forearm with TBTLF-based experiments in 200,000 episodes. (**d**) Right forearm with Bayesian-based experiments with 50 intervals.

**Figure 12:**
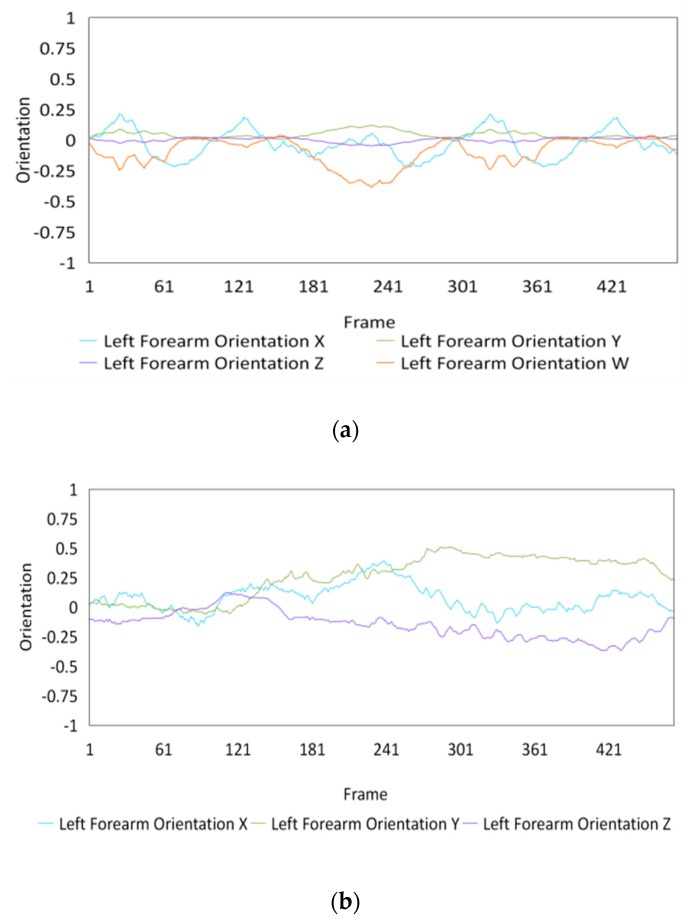

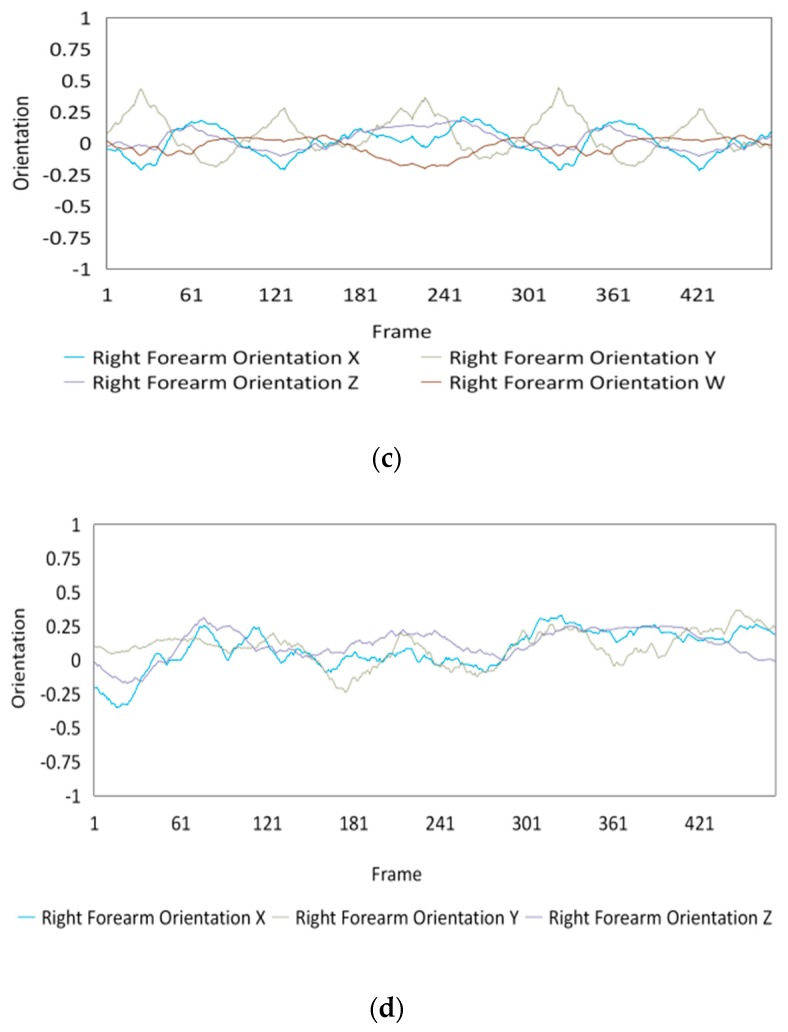
Comparison of the estimated orientations of the left arm for the 3rd subject. (**a**) Left forearm with TBTLF-based experiments in 200,000 episodes. (**b**) Left forearm with Bayesian-based experiments with 50 intervals. (**c**) Right forearm with TBTLF-based experiments in 200,000 episodes. (**d**) Right forearm with Bayesian-based experiments with 50 intervals.

The best performance by the proposed framework was achieved with 200,000 episodes, while that by Bayesian-based approach was achieved with 50 intervals. The comparisons between the two performances are illustrated in Figure 10 for Subject #1, Figure 11 for Subject #2, and Figure 12 for Subject #3. Given that only forearm (both a left and a right forearm) orientations were estimated in the Bayesian-based experiments, only the performances of the estimated movement of the forearm were compared. 

The movements estimated by the TBTLF-based experiments showed a great regularity, revealing the feature and discipline between the motions and subjects. Meanwhile, the movements estimated by the Bayesian-based experiments were chaotic; consequently, this method could not estimate the consecutive movements to show an entire motion.

**Figure 13:**
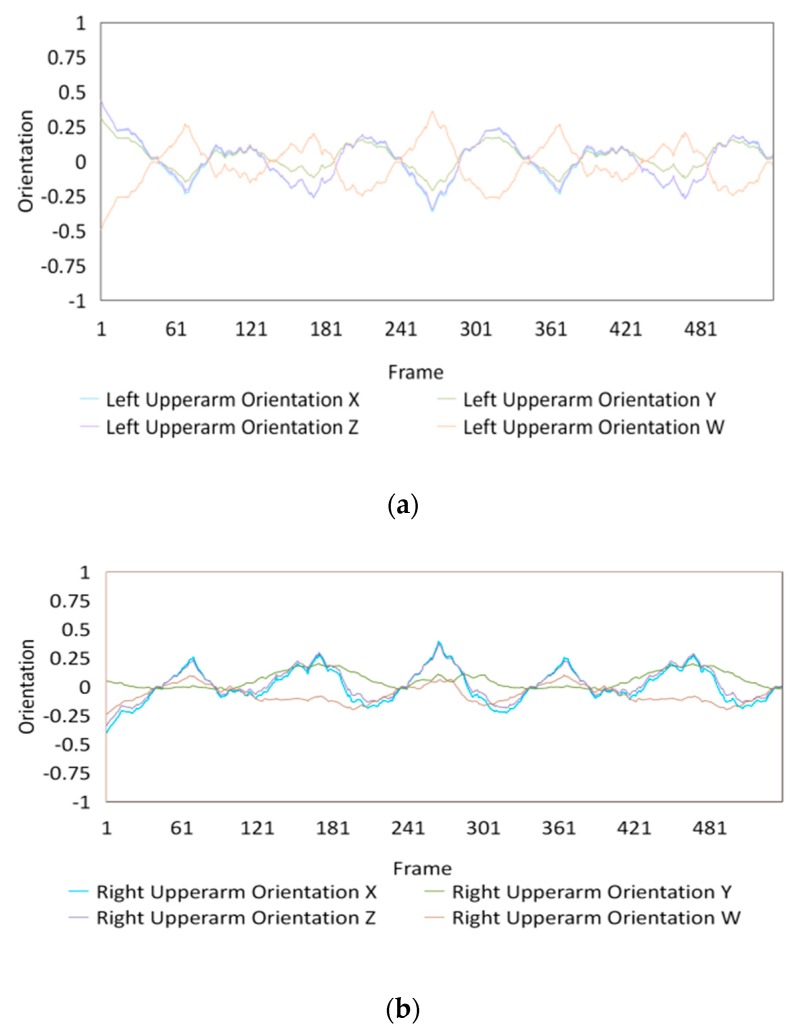
Orientations of the upper arm with TBTLF-based experiments in 200,000 episodes for the 1st subject. Estimated movements of the (**a**) Left upper arm and (**b**) Right upper arm.

**Figure 14:**
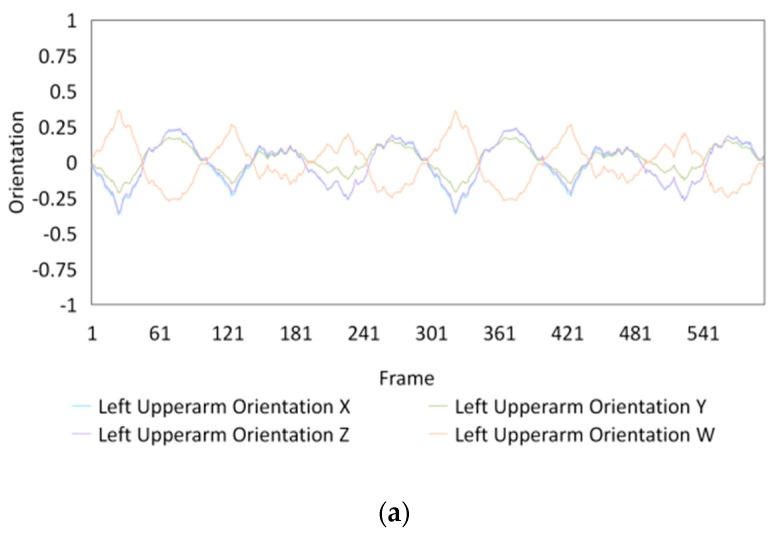

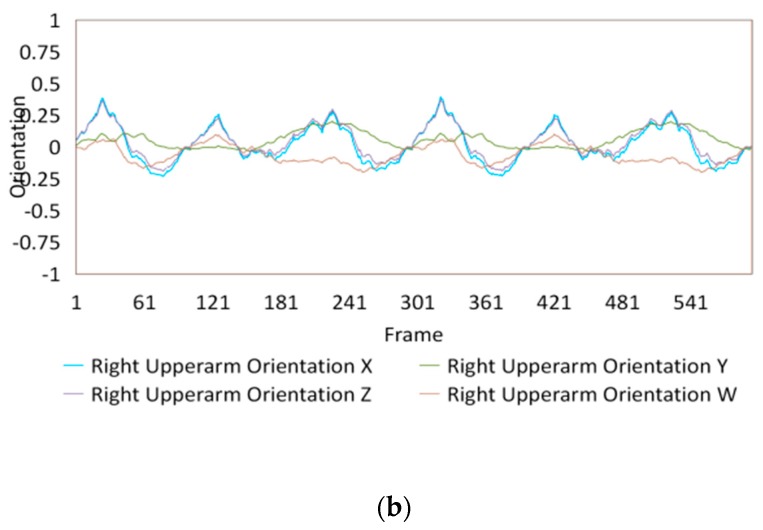
Orientations of the upper arm with TBTLF-based experiments in 200,000 episodes for the 2nd subject. Estimated movements of the (**a**) Left upper arm and (**b**) Right upper arm.

**Figure 15:**
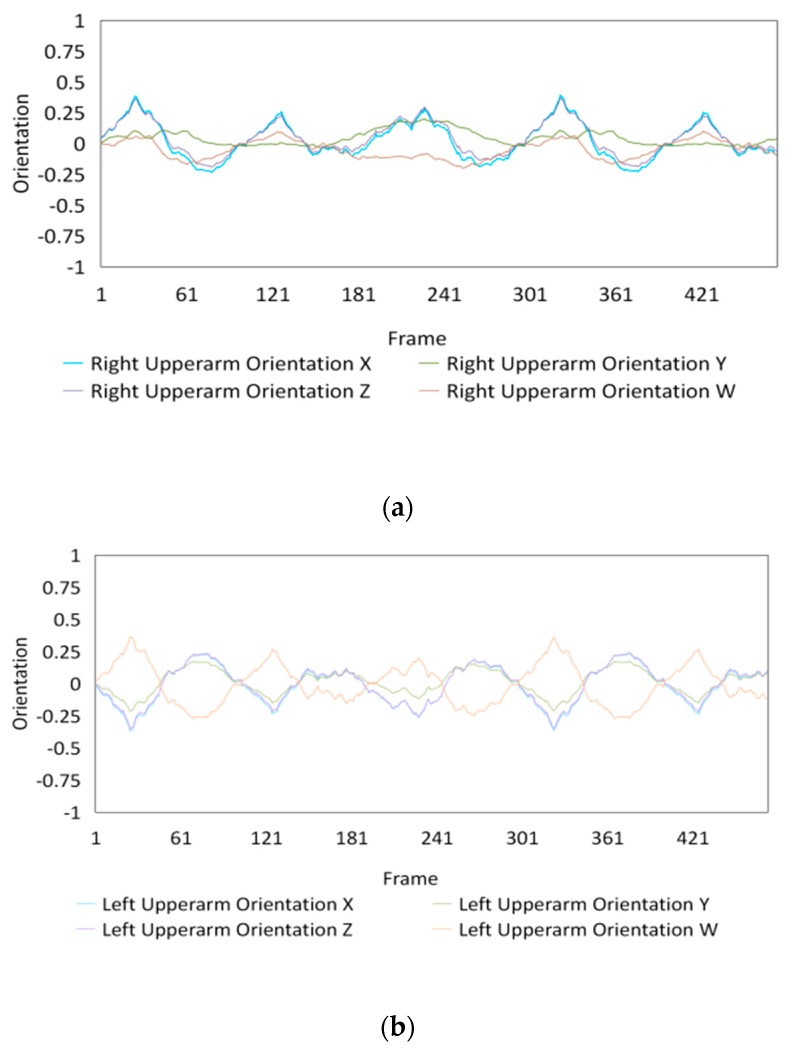
Orientations of the upper arm with TBTLF-based experiments in 200,000 episodes for the 3rd subject. Estimated movements of the (**a**) Left upper arm and (**b**) Right upper arm.

The estimated movements of the left upper arm and right upper arm for Subjects #1, #2, and #3 when TBTLF-based experiments achieved the best performance with 200,000 episodes are depicted in Figure 13, Figure 14 and Figure 15, respectively.

**Figure 16:**
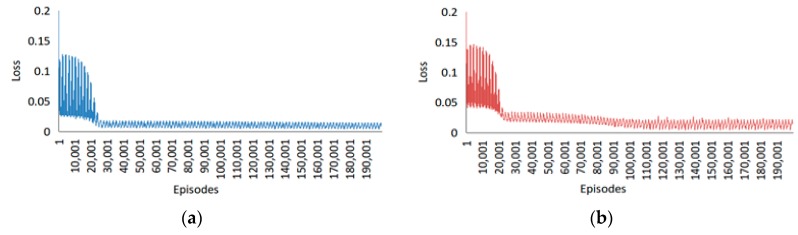
Loss during the training of the proposed framework. Loss of the (**a**) Left arm and (**b**) Right arm.

The loss of the left and right arms during the training of the proposed frameworks with 200,000 episodes are as shown in Figure 16. At first, the loss began at ~0.2; it then dropped to ~0.125. Afterward, there was a sharp decrease from 0.125 to 0.025 before 25,000 episodes for both left and right hands. Following this, a stable and slight decrease occurred until 200,000 episodes for the left hand while for the right, another slightly stronger decrease was observed from 25,000 to 110,000 episodes.

**Figure 17:**
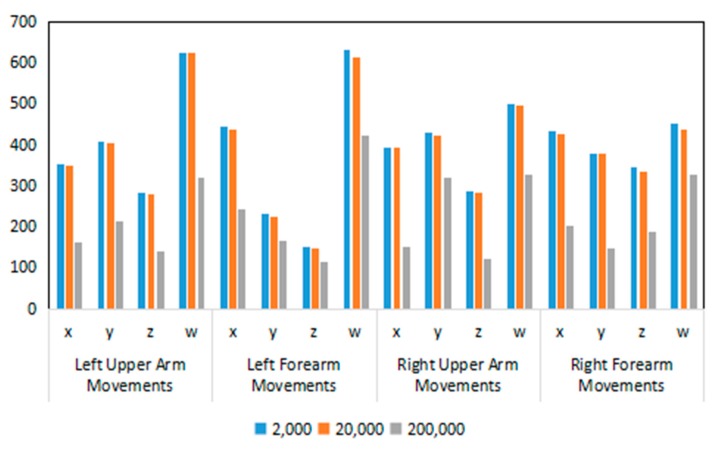
Comparison of the DTW distances for orientations x, y, z, and w in the TBTLF-based experiments.

DTW distance was used to calculate the distance among every estimated and measured arm movements to compare the similarity among them. For example, the DTW distance of the left upper arm was calculated with estimated coordinates of the left upper arm and measured coordinates of left upper arm, in which the estimated coordinates of the left upper arm were the estimated results of the proposed framework and the measured coordinates of left upper arm are the label data of the dataset. The higher the DTW distance is, the less similar the estimated movement is to the measured movement. In order to make a more intuitive comparison, the sum of DTW distances of the three subjects was used for experimental verification. The DTW distances obtained from the TBTLF-based and Bayesian-based experiments are shown in Table 4 and Table 5, respectively. Bayesian-based experiments were performed according to [11], which only estimated the x, y, and z coordinate values of the arm orientations to represent the arm movement. Figure 17 compares the DTW distances for orientations x, y, z, and w among 2000, 20,000, and 200,000 episodes in the TBTLF-based experiments.

According to Table 4 and Table 5, the best performance in the Bayesian-based experiments was obtained with 50 intervals, and that in the TBTLF-based experiments was obtained with 200,000 episodes.

The Bayesian-based framework only focuses on the x, y, and z coordinates of the forearm orientations of left and right arms. However, the proposed framework estimated x, y, z, and w coordinates of both forearm orientations of the left and right arms and the upper arm orientations of left and right arms. Consequently, the reduction rate of the DTW distance R is only calculated for the estimated forearm orientations of the left and right arms in the TBTLF-based experiment and Bayesian-based experiment, according to Equation (16). The results are given in Table 6.

(16) $$R=\frac{D^{B}-D^{T}}{D^{B}}$$

where $D^{B}$ is the DTW distance of Bayesian-based experiments and $D^{T}$ is the DTW distance of TBTLF-based experiments.

The results show that the framework proposed by us can estimate the arm orientation with an average of 73.90% reduction rate of the DTW distance compared to the traditional framework, confirming that the proposed framework can estimate movements much more accurately.

## 5. Discussion

According to the experimental results presented in Section 4, the performance of the Bayesian-based experiments remained stable and no obvious progress was observed even with more intervals, while in the TBTLF-based experiments, a significant improvement was achieved between 20,000 episodes and 200,000 episodes. That is, the performance of the TBTLF-based experiment with 200,000 episodes was found to be much better than that of any of the Bayesian-based experiment. In addition, the Bayesian-based experiments can only estimate the arm movement according to the hand movement within the range of the training data due to the limitation of the Bayesian probability. However, in the TBTLF-based experiments, the arm movement could be estimated even when the validation data was not in the range of the training data, which shows the better flexibility of the TBTLF-based movement estimation. 

## 6. Conclusions

This paper proposed a deep learning approach for human movement estimations. Firstly, movements were collected by HTC VIVE and Myo armbands, and the collected data were analyzed, wherein the movements were represented by arm orientations and hand positions. The proposed TBTLF-based framework estimated the movements of one upper arm and one forearm based on left- and right-hand movements. The TBTLF-based experiments showed significant improvements when using 200,000 episodes than when using 2000 episodes and 20,000 episodes, and also compared to the Bayesian-based experiments with 50, 100, and 1000 intervals. The effectiveness of the proposed framework was verified by several experiments, showing an average 73.90% reduction in DTW.

The proposed framework requires large amounts of training data to achieve good performance in movement estimation. Therefore, in future work, we plan to enhance the framework to reduce the size of the dataset required for accurate movement estimation.

## Figures and Tables

**Table 1:** Comparison of the previously developed frameworks with the proposed framework.

	Goal	Device	Input	Algorithm	Output
**Rahil [18]**	Estimating accurate 3D pose of human	_	Multi-view images	Deep neural network	Human 3D pose
**Arjun [19]**	Estimating the human gesture in videos with a CNN	_	3D tensor containing RGB images and their corresponding gesture features	Convolutional Neural Network	3D tensor containing response-maps for estimated 2D locations of human joints
**Hu [23]**	Detecting the surface conditions of the road and age-group of the subjects	One IMU	Signals of a single IMU device	LSTM	Surface conditions and age-group status
**Aria [20]**	Estimating the human gesture in videos with an unsupervised CNN	_	Pairs of images	Convolutional Neural Network	Dense gesture field
**Kim [15]**	Estimating one upper arm gesture depends on one forearm gesture	Two Myo armbands	Orientations of an upper arm and a forearm	Bayesian probability	Estimated upper arm gesture angles
**Lee et al. [14]**	Estimating one upper arm gesture depends on one forearm gesture	Myo armband	Orientations of an upper arm and a forearm	Bayesian probability	Estimated upper arm gesture angles
**Choi [11]**	Estimating one forearm depends on the positions of one hand	One Myo armband & one VIVE	Myo armbands: orientations of a forearm VIVE: positions of a hand	Bayesian probability	Estimated orientations of upper arm
**The proposed framework**	Estimating one upper arm and one forearm depends on the positions of two hands	Two Myo armbands & two VIVE	Myo armbands: orientations of forearms and upper arms of one arm VIVE: Positions of left and right hand	Bi-LSTM	Estimated orientations of forearms and upper arms of left and right arm

**Table 2:** Motion-based gestures.

Index	Gesture	Consecutive Motions
1	Capturing equipment	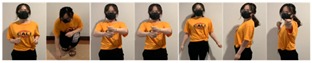
2	Fighting with wolves	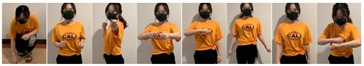
3	Searching for treasure	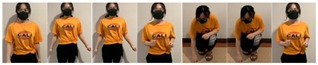
4	Going through the cave	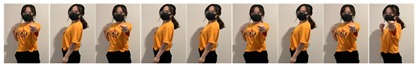
5	Getting out of the reservoir	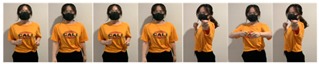
6	Exiting though the window	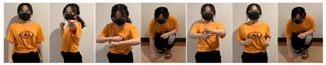
7	Exploring the cave	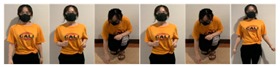
8	Running away	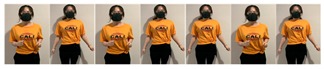
9	Through the waterfall	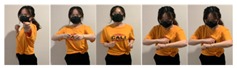
10	Through the tunnel	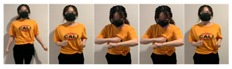
11	Robbing room	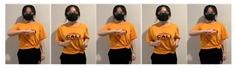
12	Forward to Mountain	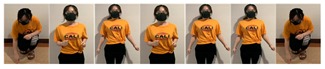
13	Climbing	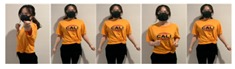
14	Attacking on the enemy	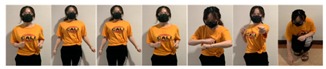
15	Fighting for survival	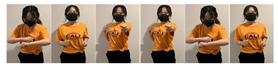

**Table 3:** Anthropomorphic information from three subjects.

	Subject #1	Subject #2	Subject #3
Gender	Female	Male	Female
Height (cm)	160	173	164
Weight (kg)	52	61	55
Length of Arms (cm)	62	70	65

**Table 4:** Dynamic time warping (DTW) distances of TBTLF-based experiments.

	Episodes	2000	20,000	200,000
Estimated Movements				
Left Upper Arm Movements	x	350.94	349.77	160.68
	y	406.34	405.38	211.71
	z	284.34	280.96	140.71
	w	624.28	622.53	319.10
Left Forearm Movements	x	443.70	437.74	241.90
	y	230.44	223.56	166.38
	z	149.74	147.14	114.36
	w	631.33	612.90	421.90
Right Upper Arm Movements	x	391.27	391.26	152.05
	y	431.06	423.72	318.30
	z	286.06	282.60	122.65
	w	500.76	495.73	328.40
Right Forearm Movements	x	434.38	426.66	201.85
	y	379.77	377.07	146.45
	z	344.16	333.24	187.36
	w	451.01	438.51	326.53

**Table 5:** DTW distances of Bayesian-based experiments.

	Intervals	50	100	1000
Estimated Gestures				
Left Forearm Movements	x	750.55	780.85	680.01
	y	681.49	708.28	751.25
	z	441.44	751.25	706.79
Right Forearm Movements	x	846.90	677.68	854.68
	y	815.04	882.20	823.23
	z	581.09	563.97	781.93

**Table 6:** Reduction rate of the DTW distance.

Estimated Movements		Reduction Rate of DTW Distance
Left Forearm Orientations	x	67.77%
	y	75.59%
	z	74.09%
Right Forearm Orientations	x	76.17%
	y	82.03%
	z	67.76%
Average		73.90%
